# Efficient Therapeutic Delivery by a Novel Cell-Penetrating Peptide Derived from Acinus

**DOI:** 10.3390/cancers12071858

**Published:** 2020-07-10

**Authors:** Justine Habault, Claire Fraser, Ewa Pasquereau-Kotula, Maëlys Born-Bony, Anne Marie-Cardine, Jean-Luc Poyet

**Affiliations:** INSERM UMRS976, Université de Paris, Institut de Recherche Saint Louis, Hôpital Saint Louis, Bâtiment Hayem, 1 Avenue Claude Vellefaux, 75010 Paris, France; justine.habault@gmail.com (J.H.); claire.frasr@gmail.com (C.F.); ewa.kotula86@gmail.com (E.P.-K.); maelys.bornbony@gmail.com (M.B.-B.); anne.marie-cardine@inserm.fr (A.M.-C.)

**Keywords:** cell-penetrating peptides, anticancer peptide, sézary syndrome, AAC-11, therapeutic target, acinus

## Abstract

In this study, we have identified a novel cell-penetrating sequence, termed hAP10, from the C-terminus of the human protein Acinus. hAP10 was able to efficiently enter various normal and cancerous cells, likely through an endocytosis pathway, and to deliver an EGFP cargo to the cell interior. Cell penetration of a peptide, hAP10DR, derived from hAP10 by mutation of an aspartic acid residue to an arginine was dramatically increased. Interestingly, a peptide containing a portion of the heptad leucine repeat region domain of the survival protein AAC-11 (residues 377–399) fused to either hAP10 or hAP10DR was able to induce tumor cells, but not normal cells, death both ex vivo on Sézary patients’ circulating cells and to inhibit tumor growth in vivo in a sub-cutaneous xenograft mouse model for the Sézary syndrome. Combined, our results indicate that hAP10 and hAP10DR may represent promising vehicles for the in vitro or in vivo delivery of bioactive cargos, with potential use in clinical settings.

## 1. Introduction

The poor permeability and selectivity of the cell membrane strongly limit the repertoire of possible pharmaceutical agents and biologically active molecules. Established methods for delivery of cell-impermeable materials, such as viral vectors and membrane perturbation techniques, suffer a number of limitations, such as inefficiency, cytotoxicity, or lack of reliability for in vivo settings [1,2]. Consequently, in recent years, much effort has been dedicated towards developing novel strategies allowing the intracellular delivery of bioactive cargos into live cells. Cell-penetrating peptides (CPPs), also known as protein transduction domains (PTDs), are a class of short (less than 30 residues), cationic, and/or amphipathic peptides which has been extensively shown to be capable of translocating though various biological membranes via direct penetration and/or endocytosis [3,4,5,6]. Over the past few years, CPPs have received significant attention as delivery agents for a wide range of cargos, such as proteins, peptides, DNAs, siRNAs, nanoparticles, and small chemical compounds both in vitro and in vivo [7,8,9,10,11]. Applications include both fundamental biology, such as the transport of fluorescent or radioactive agents for imaging purposes, stem cell manipulation, and reprogramming and gene editing [12,13,14,15,16], as well as preclinical and clinical trials to investigate medical applications of CPP-derived therapeutics against various diseases, including heart disease, stroke, cancer, and pain [7,17]. Most of the CPPs in use today are pathogen-derived or synthetic entities and therefore feature the potential risk of immunogenicity and cytotoxicity, especially when conjugated to a protein or nanoparticle, restricting their use for biomedical applications [7,17]. Moreover, many described CPPs exhibit low delivery efficiency. Consequently, the development of novel human originated CPPs with a high transduction efficiency is of great interest. In this study, we have identified and characterized a novel cell-penetrating sequence, termed hAP10, from the C-terminus of the human protein Acinus and evaluated its potential as a cargo protein carrier. A peptide derived from hAP10 by the mutation of an aspartic acid residue to an arginine, called hAP10DR, was also developed and its penetrating properties explored. Finally, the antitumor effect of hAP10 or hAP10DR conjugation with the protein–protein interaction domain of the survival protein AAC-11 was investigated in vitro and in vivo in a sub-cutaneous xenograft mouse model for the Sézary syndrome.

## 2. Results

### 2.1. Acinus Contains a CPP-Like Sequence

**Figure 1:**
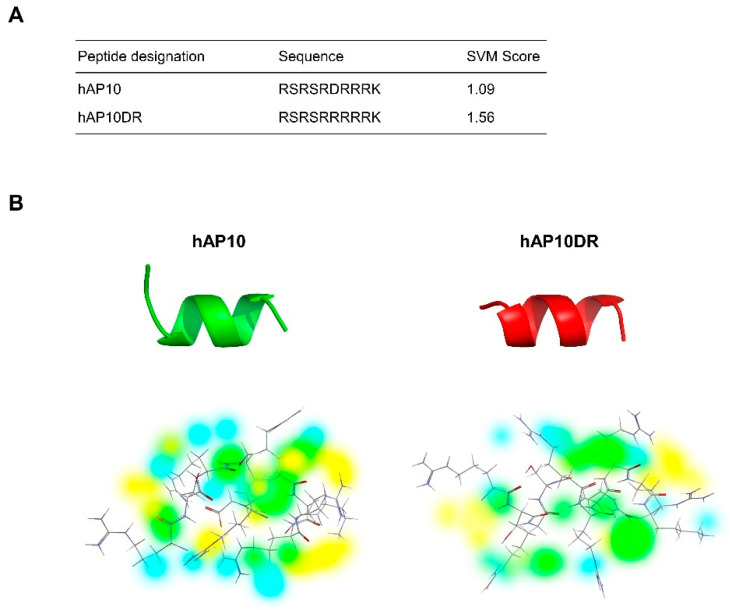
Sequence and structural prediction of the investigated peptides. (**A**) Name, amino-acid sequences and support vector machine (SVM) score of the potential CPPs. The SVM-based method, which uses binary profile of the peptide, was used for the SVM score prediction. (**B**) Top: Structural prediction of hAP10 and hAp10DR. Bottom: Energy maps of hAP10 and hAP10DR. Coloring is the following: hydrogen donor favorable (yellow), hydrogen acceptor favorable (blue) and steric favorable (green).

In exploring the sequence of Acinus (Apoptotic chromatin condensation inducer in the nucleus), a nuclear protein involved in in RNA processing and apoptotic DNA fragmentation [18,19,20,21,22,23], we noticed an arginine rich region located in the C-terminus that presents significant similarities with the sequence of the TAT CPP (residues 1177–1186 of Acinus-L, Figure 1A). Analysis of these 10 residues sequence, hereafter called hAP10, using the CellPPD in silico tool confirmed that hAP10 could indeed possess CPP properties (Figure 1A).

Cationic CPPs have a net positive charge at physiological pH, mostly derived from arginine and lysine residues in their sequence, which drives their cell-penetrating properties [7]. hAP10 is highly cationic with six arginine and one lysine residues. As it contains one aspartic acid at its center, and because replacing negative charged residues with positively charged residues can increase penetrating activity of cationic CPPs, we hypothesized that substitution of hAP10 aspartic acid to an arginine (hAP10DR) could potentially increase its penetrating properties. Indeed, as shown in Figure 1A, in silico analysis using the online prediction tool CellPPD (http://crdd.osdd.net/raghava/cellppd/) resulted in a higher support vector machine (SVM) score for hAP10DR compared to hAP10. The secondary structure of CPPs are important for their membrane interaction and it has been shown that peptides with an α-helical region can internalize more efficiently than their random-coiled counterparts [24]. Secondary and three-dimensional structure predictions carried out with the well-established PSIPRED and I-TASSER servers [25,26] suggested an essentially helical structure for both hAP10 and hAP10DR, with an helical content of 70% and 80%, respectively (Figure 1B). Energy maps revealed slightly different favorable hydrogen donor and acceptor regions and steric fields around the peptides (Figure 1B). As these observations suggest that hAP10 and hAP10DR could represent novel CPPs, both peptides were selected for experimental validation and further analysis of their in vitro and in vivo cargo delivery properties.

### 2.2. Cellular Uptake of hAP10 and hAP10DR

**Figure 2:**
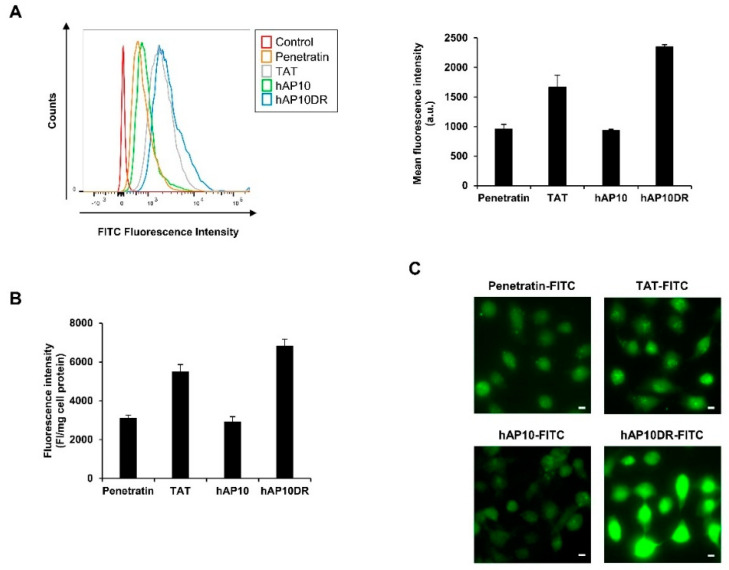
Cellular uptake of hAP10 and hAP10DR. (**A**) HUT78 cells were incubated with 5 µM of FITC-labelled hAP10 and hAP10DR or penetratin and TAT as controls for 1 h in complete medium. Cells were then washed with PBS, incubated in trypsin-EDTA solution (0.01% trypsin) at 37 °C for 10 min, resuspended in PBS and subjected to flow cytometry (right). Left: Bar diagram representing the uptake of the FITC-labelled peptides as mean cellular fluorescence from the flow cytometry analysis of live cells positive for FITC. Data are means ± s.e.m. (*n* = 3). (**B**) Fluorescence quantification of FITC-labelled hAP10 and hAP10DR uptaken in human B lymphocytes. Cells were incubated with 5 µM of FITC-labelled hAP10 and hAP10DR or penetratin and TAT as controls for 1 h in complete medium, washed with PBS and the fluorescence of the cell lysis measured as described in Material and Methods. Data are means ± s.e.m. (*n* = 3). (**C**) (Bottom-right figure) Intracellular distribution of FITC-labelled hAP10 and hAP10DR in U2OS cells. U2OS cells grown on coverslips were incubated with 5 µM of FITC-labelled hAP10 and hAP10DR or penetratin and TAT as controls for 1 h in complete medium, washed trice with PBS and live cells were imaged using fluorescence microscopy. All images were acquired using the same light intensity and microscope settings to permit direct comparison between the peptides. The scale bar indicates 10 µm.

The cell uptake efficacy of FITC-labeled hAP10 and hAP10DR was first assessed by flow cytometry analysis and compared to that of the widely used CPPs penetratin and TAT. Cellular uptake was analyzed after 60 min incubation of HUT78 Sézary cells and stringent washing followed by incubation with trypsin to remove the extracellular membrane-associated peptides [5]. As shown in Figure 2A, both hAP10 and hAP10DR were efficiently internalized into HUT78 cells.

Importantly, hAP10 displayed a similar uptake efficiency to that of penetratin. hAP10DR however, showed a higher uptake and was internalized approximately twice as more efficiently than its wild type counterpart and about 50% more than TAT (Figure 2A), indicating that replacement of the negatively charged aspartic acid with the positively charged arginine drastically favored the CPP capacities of the peptide. Similar data were obtained using U2OS and C8161 cancer cells (Supplementary Figure S1). Interestingly, hAP10 and hAP10DR were able to permeate into non-cancerous cells, such as human B lymphocytes (Figure 2B). We next examined the cellular distribution of hAP10 and hAP10DR using fluorescent microscopy imaging. U2OS cells were treated with FITC-labeled hAP10 and hAP10DR or the control peptides penetratin and TAT and the cells were imaged using live microscopy imaging. We chose to perform these experiments on live cells to avoid fixation artefacts that can arise when studying transduction of arginine-rich peptides As shown in Figure 2C, both hAP10 and hAP10DR as well as the control peptides adopted both a punctuate but mostly diffuse fluorescence distribution throughout the cells, confirming that the peptides were indeed internalised and not merely adsorbed at the cell surface. In agreement with the cytometry profiles, the intracellular fluorescence intensity of the hA10DR peptide was much higher to that of hAP10 and control peptides penetratin and TAT, confirming the superior transduction efficacy of the mutated version of the peptide.

### 2.3. Cellular Uptake Mechanism of hAP10 and hAP10DR

**Figure 3:**
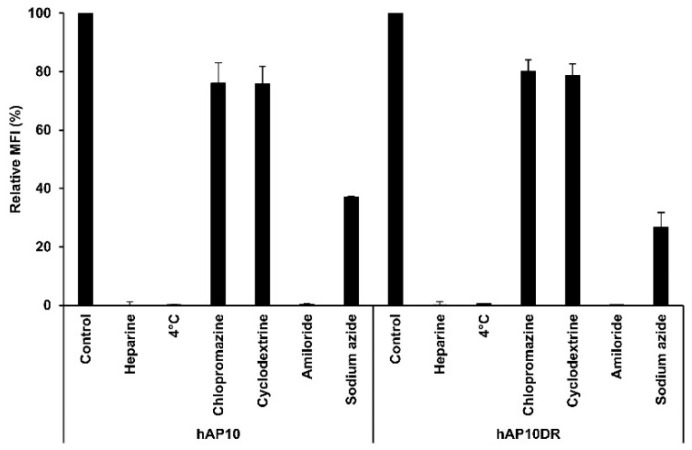
Internalization mechanisms of hAP10 and hAP10DR. C8161 cells pre-incubated at 4 °C or with heparin sulfate (20 μg/mL), sodium azide (0.1%), CPZ (50 µM), MBCD (1 mM) or EIPA (50 µM) for 30 min or left untreated were incubated with 5 µM of FITC-labelled hAP10 and hAP10DR for 1 h in complete medium. Cells were then washed with PBS, detached with trypsin, washed and suspended in PBS, then subjected to flow cytometry (left). Right: Bar diagram representing the uptake of the FITC-labelled peptides as mean cellular fluorescence from the flow cytometry analysis of live cells positive for FITC. Data are means ± s.e.m. (*n* = 3).

Although the precise mechanisms by which CPPs enter the cells are still under debate, they fall into two broad categories: direct translocation and endocytosis [7]. To gain insight into the transduction process of hAP10 and hAP10DR, we investigated the effect of heparin, temperature and well-established endocytosis inhibitors on the cellular uptake of hAP10 and hAP10DR. As shown in Figure 3, cellular uptake of both hAP10 and hAP10DR into C8161 cells was greatly decreased in the presence of heparin sulfate, indicating that the peptides penetrate the membrane via heparin sulfate proteoglycan (HSPG)-mediated pathway(s). Similar data were obtained using U2OS cells (Supplementary Figure S2). We next tested whether the cellular internalization of hAP10 and hAP10DR was mediated by an energy-dependent process. As endocytosis is form of active transport, requiring energy, lowering the temperature is expected to inhibit endocytic processes but not energy-independent processes such as direct penetration. As shown in Figure 3, the cellular uptake of hAP10 and hAP10DR was substantially decreased when exposed cells were incubated at 4 °C as compared to 37 °C. Similar results were observed following energy depletion by sodium azide (Figure 3). Combined, these data indicate that hAP10 and hAP10DR are internalized into cells through an energy-dependent endocytosis mechanism. We next evaluated the precise cell entry pathway of hAP10 and hAP10DR by using various inhibitors of known endocytic pathways. Pre-treatment of cells with chlorpromazine (CPZ), a known inhibitor of clathrin-mediated endocytosis, or methyl-β-cyclodextrine (MBCD), an inhibitor of lipid raft-mediated endocytosis, did not significantly reduce the uptake of hAP10 and hAP10DR (Figure 3). However, a drastic decrease was observed upon pre-treatment of the cells with 5-(N-ethyl-isopropyl) amiloride (EIPA), an inhibitor of micropinocytosis (Figure 3). Similar data were obtained when using U2OS cells (Supplementary Figure S2). Together, these results identify macropinocytosis as the main pathway for hAP10 and hAP10DR cellular uptake.

### 2.4. Analysis of Cellular Toxicity, Hemolytic Activity and Immunogenicity of hAP10 and hAP10DR

**Figure 4:**
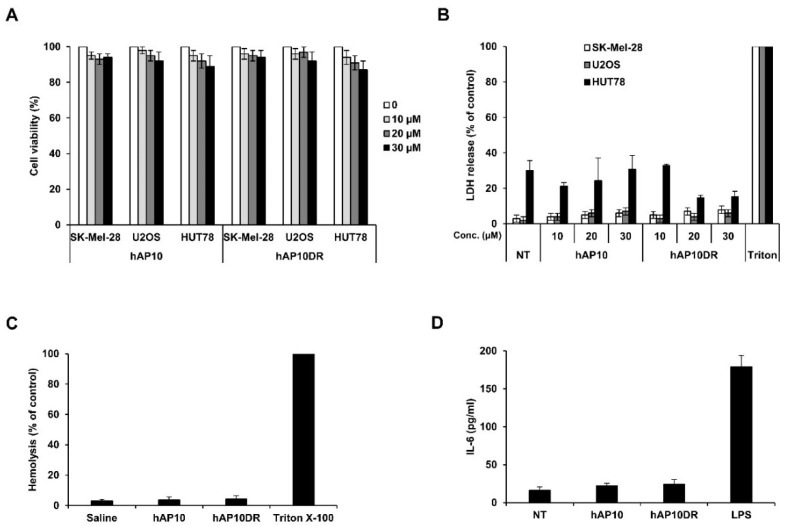
Lack of toxicity and immunogenicity of hAP10 and hAP10DR. (**A**) The indicated cells were exposed to increasing concentrations of hAP10 or hAP10DR for 20 h. Viability was then assessed by an MTT assay. Data are means ± s.e.m. (*n* = 3). (**B**) Necrotic cell death was monitored by lactate dehydrogenase (LDH) release from cells into the culture medium. The obtained values were normalized to those of the maximum LDH released (completely lysed) control. Data are means ± s.e.m. (*n* = 3). (**C**) hAP10 and hAP10DR do not induce hemolysis in vitro. Mice red blood cells were incubated with 30 μM of hAP10 or hAP10DR. Released hemoglobin was detected by densitometry at 540 nm. Hemoglobin release by cells treated with 1% Triton X-100 was used as 100% lysis control. (**D**) Levels of IL-6 secretion from RAW 264.7 cells exposed to 10 µM of hAP10 or hAP10DR or LPS (1 µg/mL) for 24 h. Data are means ± s.e.m. (*n* = 3).

Similarly to other drug delivery systems, cytotoxicity and the tendency to induce innate immunity may limit CPPs uses in clinics. We first assayed the cytotoxicity effect of hAP10 and hAP10DR on various cell lines. Dose-response analyses indicate that neither peptide significantly altered cellular viability at doses up to 30 µM (Figure 4A). Moreover, the absence of lactate dehydrogenase (LDH) activity release in the culture medium indicated that hAP10 and hAP10DR did not induce membrane disturbance (Figure 4B). In line with this observation, neither hAP10 nor hAP10DR exhibited hemolytic activity (Figure 4C), confirming that the peptides do not cause membrane damage. We next evaluated the potential immunogenicity of hAP10 and hAP10DR by measuring the secreted levels of IL-6 upon treatment of RAW 264.7 mouse macrophage cells with the peptides for 24 h. Whereas the control bacteria-derived lipopolysaccharide (LPS) elicited a potent cytokine response, no significant IL-6 release was detected in the media of RAW 264.7 cells cultured in the presence of hAP10 or hAP10DR (Figure 4D). Combined, our data indicate that hAP10 or hAP10DR are essentially not cytotoxic and non-immunogenic and therefore demonstrate potential for in vivo applications.

### 2.5. Intracellular Delivery of hAP10- and hAP10DR-GFP Fusion Protein

**Figure 5:**
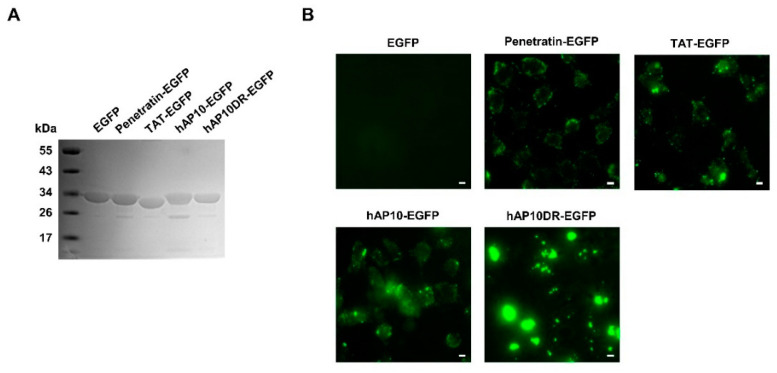
hAP10 and hAP10DR-mediated delivery of a GFP cargo into cells. (**A**) Electrophoretic analysis of the recombinant GFP derivatives. Samples (10 μg) of the indicated purified recombinant proteins were resolved by SDS-polyacrylamide gel electrophoresis followed by Coomassie Brilliant Blue staining. (**B**) U2OS cells were exposed to the indicated GFP fusion proteins (5 µM) for 1 h. Cells were then washed with PBS and live cells were imaged using fluorescence microscopy. All images were acquired using the same light intensity and microscope settings to permit direct comparison between the peptides. The scale bar indicates 10 µm.

We next evaluated the potential of hAP10 and hAP10DR to carry a functional macromolecule into cells. For that purpose, we generated recombinant fusion proteins comprising EGFP fused at the N-terminus to hAP10 or hAP10DR or the control CPPs TAT and penetratin (Figure 5A). The resulting proteins were then administered to the culture media of U2OS cells and the cells were imaged using live microscopy imaging. As shown in Figure 5B and Supplementary Figure S3, a punctate fluorescence pattern was observed for the fusion proteins but not for EGFP alone. Interestingly, in line with the FITC-labeled peptide uptake, hAP10-EGFP fluorescence was at least comparable to that of TAT-EGFP or penetratin-EGFP, whereas hAP10DR-EGFP fluorescence appeared more intense. Taken together, our data indicate that the hAP10 and mutated sequences possess strong cell penetrating activities and are at least as effective as the commonly used TAT and penetratin CPPs at delivering an EGFP cargo to the cell interior.

### 2.6. Anti-Tumoral Effect of AAC-11 Heptad Leucine Repeat-Derived Peptides

**Figure 6:**
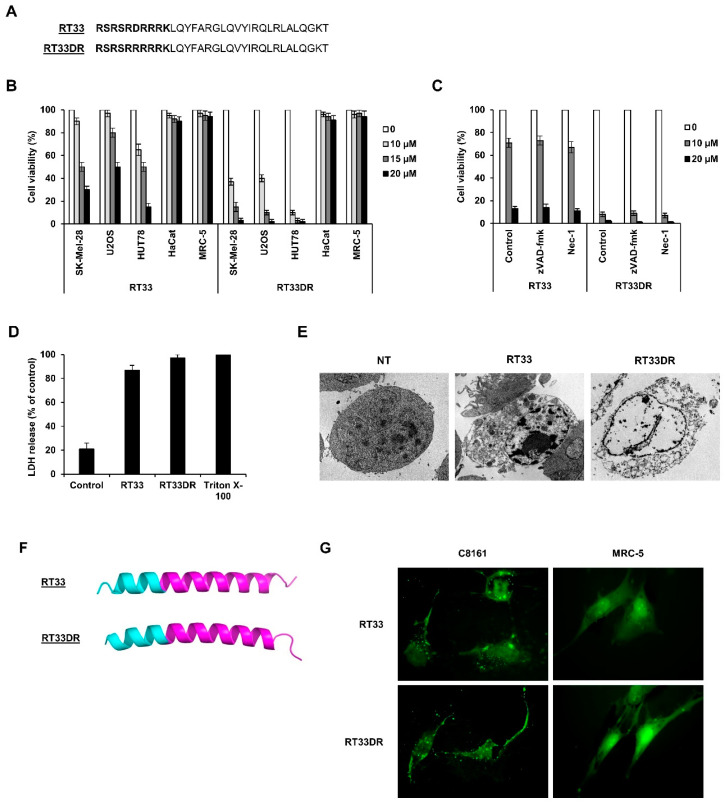
RT33 and RT33DR induces cancer cells, but not normal cells, death. (**A**) Amino-acid sequence of RT33 and RT3DR. hAP10 and hAP10DR sequences are in bold. (**B**) The indicated cells were exposed to increasing concentrations of RT33 or RT3DR for 20 h. Viability was then assessed by an MTT assay. Data are means ± s.e.m. (*n* = 3). (**C**) HUT78 cells were exposed to increasing concentrations of RT33 or RT3DR for 20 h in the presence and absence of 50 μM zVAD-fmk or 50 μM Necrostatin-1 (Nec-1). Viability was then assessed by an MTT assay. Data are means ± s.e.m. (*n* = 3). (**D**) HUT78 cells were exposed to 20 µM of RT33 or RT33DR for 3 h. Necrotic cell death was monitored by lactate dehydrogenase (LDH) as in Figure 4B. Data are means ± s.e.m. (*n* = 3). (**E**) Ultrastructural analysis of HUT78 cells untreated (NT) or treated with 15 µM of hAP10 or hAP10DR for 1 h. (**F**) Structural prediction of RT33 and RT33DR. The segments corresponding to the hAP10 and hAP10DR moieties are in magenta. (**G**) C8161or MRC-5 cells were exposed to FITC-labelled RT33 or RT33DR for 1 h. Cells were then examined by fluorescence microscopy.

We have previously reported that a penetrating peptide (peptide RT53) spanning the heptad leucine repeat region of the survival protein AAC-11 (residues 363–399) fused to the CPP penetratin induces cancer cell death in vitro and inhibits melanoma tumor growth in a xenograft mouse model [27]. We here hypothesized that a peptide comprising a smaller portion of the heptad leucine repeat region of AAC-11 attached to hAP10 or hAP10DR might possess interesting anti-cancer properties. We therefore tested the anti-tumor effects of shorter peptides containing AAC-11 residues 377–399 attached to the C-terminus of hAP10 or hAP10DR (RT33 and RT33DR peptides, respectively). To study the anticancer properties of the developed peptides, we first assessed the viability of various cancer (SK-Mel-28, U2OS, HUT78) or normal (HaCat, MRC-5) cells following exposure to increasing concentration of RT33 or RT33DR. As shown in Figure 6A, both peptides inhibited cell viability in all cancer cells in a dose-dependent manner, while sparing the normal cells tested. Of note, RT33DR exhibited substantially higher anticancer proprieties than RT33, maybe due to the high cell penetration capacity of its CPP. Neither the shuttles (Figure 4) nor the AAC-11 specific portion alone (Supplementary Figure S4) decreased cell viability, indicating that the integrity of the peptides is required for their anti-tumoral effects. We next sough to investigate RT33 and RT33DR mechanisms of cancer cell death. We were especially interested in the response of HUT78 Sézary cells because effective therapeutic options for Sézary syndrome, an erythrodermic form of cutaneous T-Cell lymphoma (CTCL), are scarce [28]. Pharmacological inhibition of the apoptotic pathways with the pan-caspase inhibitor zVAD-fmk did not block RT33 or RT33DR-induced cytotoxicity (Figure 6B), suggesting that the observed cell death does not depend on apoptosis. Furthermore, cell death was not prevented by the RIPK1 kinase inhibitor necrostatin-1, excluding necroptosis as cell death mechanism (Figure 6C). Similar data were obtained using U2OS cells (Supplementary Figure S5). In previous studies, we found that RT53 induces tumor cell necrosis [27]. We therefore assessed LDH activity release in the culture medium of HUT78 cells treated with RT33 and RT33DR. As shown in Figure 6D, peptides exposure resulted in a massive release of LDH into HUT78 treated cells supernatant, indicative of membrane lysis and necrotic cell death. Transmission electron microscopy micrographs further supported that RT33 and RT33DR induce tumor cell necrosis. In contrast, control cells showed a typical intact plasma.

Membrane HUT78 cells treated with RT33 and RT33DR exhibited ruptured and disintegrated plasma membranes, with a total loss of membrane structure (Figure 6E). In line with our precedent results (Figure 6C), no evidence of chromatin condensation was observed, indicating the RT33- and RT33DR-mediated cell death does not involve a direct form of conventional apoptosis but rather a membranolytic mode of action. Combined, our data indicate that like RT53, RT33 and RT33DR induce necrosis of cancerous cells. The ability of RT33 and RT33DR to induce plasma membrane leaking suggests that both peptides target the plasma membrane. Previous data obtained with RT53 suggested that, in analogy with pore-forming toxins, its membranolytic property was a consequence of its accumulation at the plasma membrane of cancerous cells, leading to the formation of pores and subsequent necrosis [27]. In this mechanism, the cell-penetrating moiety of RT53 allows its plasma membrane penetration, where it can bind to a membrane protein partner through its AAC-11 sequence. Local accumulation of the peptide would then lead to pores formation, owning to its alpha helical membrane active structure [27]. Structure prediction indicated that, like RT53, RT33 and RT33DR should essentially adopt an α-helical structure (Figure 6F). To provide evidence that RT33 and RT33DR target the plasma membrane, we incubated the cancerous C8161 or the not cancerous MRC-5 cells with FITC-labeled peptides and observed the fluorescence pattern. We chose C8161 cells as they are adherent and provide a big cytoplasm, which makes this cell line appropriate for imaging. As shown in Figure 6G, RT33− and RT33DR-treated cells showed punctate fluorescence over the cell surface, indicating that the peptides accumulate both at the plasma membrane and at the intracellular level. However, no RT33 or RT33DR fluorescence was observed in the membranes of the MRC-5 cells. Combined, our results strongly suggest that RT33 and RT33DR, owning to the cell-penetrating properties of the hAP10 and hAP10DR shuttles, can insert into cancer cells plasma membrane where the peptides, upon binding to a membrane-interacting partner, induce pore formation, as witnessed for RT53.

### 2.7. RT33 and RT33DR Induce Targeted Killing of Circulating Malignant T Cells in Sézary Patients’ Primary PBMC

**Figure 7:**
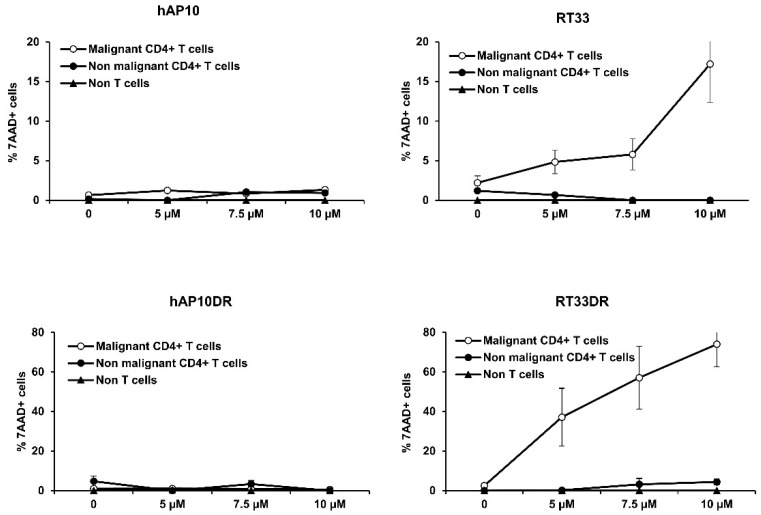
RT33 and RT33DR specifically induce primary Sézary cells death. Sézary patients’ PBMC were incubated with increasing concentrations of the indicated peptides for 4h at 37 °C. Cells were then analyzed by flow cytometry following labeling with anti-TCRVβ-FITC, −CD4−PE, −CD3−PE−Cy7 mAbs and 7-AAD. Data are shown as the means ± s.e.m. of the percentage of 7-AAD+ apoptotic cells within the following populations: malignant (CD3^+^CD4^+^TCR-Vβ^+^) and non-malignant (CD3^+^CD4^+^TCR-Vβ^−^) CD4^+^ T-cells and non T-cells (CD3^−^), derived from three different patients.

We next tested the anti-tumor effect of RT33 and RT33DR against primary Sézary cells. For that purpose, an ex vivo assay was established in which RT33 or RT33DR were directly incubated with peripheral blood mononuclear cells (PBMC) from Sézary patients. The viability of three different cell populations was then assessed by flow cytometry through the incorporation of 7-AAD: the malignant T-cell clone (Sézary cells), defined as CD3^+^CD4^+^Vβ^+^ cells, the non-malignant CD4^+^ T-cells, defined as CD3^+^CD4^+^Vβ^−^ cells, and the non T-cells, defined as CD3^−^ cells. In line with our previous data, the hAP10 or hAP10DR shuttles did not induce cell death in the transformed or normal primary cell populations (Figure 7, left), confirming their safety profile as carrier. However, both RT33 and RT33DR exhibited dose-dependent cell death activity in the malignant CD4^+^ T-cells, with RT33DR being the most efficient peptide toward Sézary cells (Figure 7, right). Strikingly, neither peptide decreased cell viability of the non-tumoral CD4^+^ T-cell as well as non-T-cell populations, even at the highest doses. Therefore, these results demonstrate that RT33 and RT33DR selectively induce primary Sezary cells death in a dose-dependent manner, without harming primary normal cells, indicating that the peptides possess a cancer cell selective killing property.

### 2.8. RT33 and RT33DR Induce Tumor Growth Reduction in a Xenograft Murine Model of Sézary Syndrome

**Figure 8:**
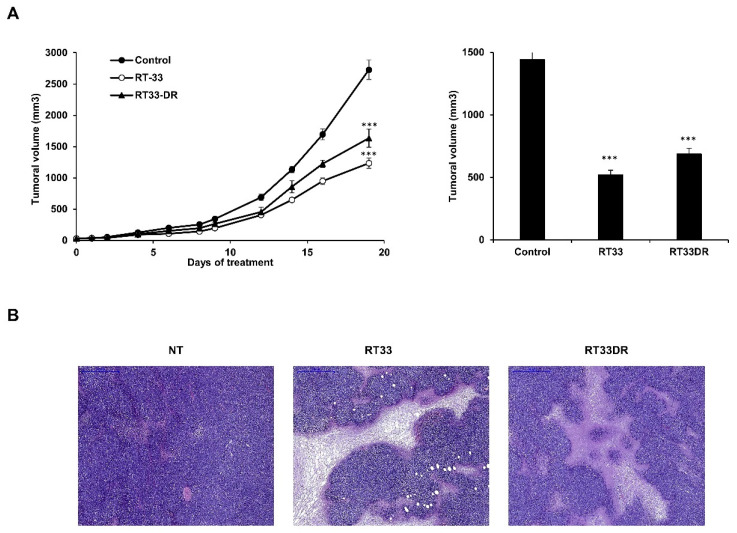
RT33 and RT33DR inhibit tumor growth in vivo in a mouse model for the Sézary syndrome. (**A**) Mice were engrafted subcutaneously with HUT78 Sézary cell line. Animals with preexisting tumors were treated daily with i.p. injections of RT33 or RT33DRM in normal saline (5 mg/kg) or normal saline as control. Tumors were calipered throughout the study and data were plotted as means ± s.e.m. (*n* = 7 mice per group). *** *p* < 0.005 relative to control. Subsequently, tumors were excised, stripped of non-tumor tissue and tumors volumes were calculated. (**B**) Representative pictures of H&E staining of tumors treated with RT33, RT33DRM, or normal saline. The scale bar represents 500 µm.

To assess in vivo antitumor activity of RT33 and RT33DR, HUT78 Sézary cells were inoculated subcutaneously to NOD/SCID gamma (NSG) mice. When the xenografted tumors reached a volume of approximately 100 mm^3^, mice were randomized and injected daily with normal saline (NT) or 5 mg/kg of RT33 or RT33DR peptides. No obvious clinical symptoms were observed during the experimental period with either peptide. As shown in Figure 8A (left), both peptides induced significant tumor growth reduction as compared to control mice, with approximate tumor growth reduction of 66% (*p* < 0.005) for RT33 and 60% for RT33DR. Similarly, upon sacrifice at the study end point, xenograft tumors were excised and stripped of non-tumor tissue, if present, for more precise ex vivo measurement. As shown in Figure 8A (right), the total tumor volume was decreased more than 2.6 times in RT33 treated mice and more than two-fold in RT33DR treated mice as compared with that in control mice. Assessment of tumor necrosis by H&E staining revealed a sharp increase of necrotic areas in RT33 or RT33DR treated groups compared to the control group (Figure 8B). Combined, these data indicate that both RT33 and RT33DR are well tolerated in vivo and can reduce tumor growth as single agents upon systemic administration by inducing tumor cells necrosis.

## 3. Discussion

Here, we identified and characterized a new CPP corresponding to residues 1177–1186 of human Acinus-L, termed hAP10, as well as its derivative hAP10DR. In vitro approaches demonstrated that hAP10 displayed excellent cell penetration efficiencies in both normal and cancerous cells, equaling classical CPPs such as TAT and penetratin while being shorter. Previous studies have demonstrated that the guanidium group of arginine is critical for cationic CPPs activity, through interaction with negatively charged components of membranes, and the number of arginines present in a sequence affects internalization efficiency [29,30,31]. Interestingly, we observed remarkably augmented cell penetration efficiency of the hAP10DR derivative, in which we replaced the negatively charged aspartic acid present in the wild type counterpart with an arginine, as hAP10DR largely outperformed hAP10 as well as TAT and penetratin. Among other parameters, the cell penetration properties of CPPs are also dependent of their secondary structure and it has been shown that peptides with a α-helical region can more efficiently enter cells [32,33]. hAP10 and hAP10DR mostly adopt a helical structure, which can therefore explain their interesting CPP properties. Importantly, neither hAP10 nor hAP10DR induced membrane disturbance or detectable cellular toxicity. Both peptides are also non-immunogenic, making them attractive and safe carriers for in vivo applications. Biochemical investigations revealed the involvement of a heparan sulfate proteoglycan-mediated micropinocytosis as a major route of internalization for hAP10 and hAP10DR. Still, as multi-endocytic routes are often involved in CPPs uptake, further studies would be needed to clarify the exact internalization mechanisms for hAP10 and hAP10DR. To further evaluating the potential of hAP10 and hAP10DR as macromolecules delivery tools, the peptides were firstly conjugated with GFP. Both hAP10-GFP and hAP10DR-GFP fusion proteins were efficiently transduced in cultured cells, demonstrating hAP10 and hAP10DR interest as novel vehicles for intracellular protein delivery. Of note, hAP10DR was a far better carrier than TAT or penetratin for GFP intracellular delivery, in line with its superior penetrating ability. Finally, we evaluated the performances of hAP10 and hAP10DR through the design and study of tumor targeting peptides. Our previous studies showed that inhibiting interactions between the survival protein AAC-11 and its binding partners drastically increased the susceptibility of tumor cells to apoptosis [22]. A cell penetrating peptide (RT53) based on the fusion of penetratin and the heptad leucine repeat region of AAC-11 (residues 363–399), which functions as a protein–protein interaction module, was shown to induce cancer cell death in vitro and to inhibit melanoma tumor growth in a xenograft mouse model [27,34]. We hypothesized here that a peptide similar to RT53 but based on hAP10 and hAP10DR CPPs might possess valuable anti-cancer properties. The heptad leucine repeat region of AAC-11 is encoded by two exons (exons 9 and 10). As exons often correspond to structural and functional units of a protein, one can envision that only one of the two exons encoding AAC-11 heptad leucine repeat region could carry the anticancer activity exhibited by the RT53 peptide, making it possible to shorten the AAC-11 specific domain of the peptide. Our previous work indicated that mutation of two exon 10-encoded leucine residues in RT53 (corresponding to positions 384 and 391 of AAC-11), that were identified as critical for AAC-11 scaffolding and anti-apoptotic function [22,35], abrogated RT53 anti-tumor activity [27]. We therefore designed two peptides, designed RT33 and RT33DR, consisting of AAC-11 residues 377-399, that are encoded by exon 10, attached to the C-terminus of hAP10 or hAP10DR, respectively, and tested their anticancer properties. Interestingly, both peptides were able to selectively kill cancer cells in vitro, without affecting normal cells. Our results show that RT33- and RT33DR-induced cancer cells death occurred through an apoptosis-independent, membranolytic mechanism, as evidenced by LDH release assays as well as electron microscopy results. Like RT53, RT33 and RT33DR accumulate at the plasma membrane level of cancer cells, but not of non-cancerous cells. Even the known contribution of the physico-chemical properties of tumor cells membranes cannot formally be excluded, as we hypothesize that RT33 and RT33DR, as witnessed with other cancer cell-specific, membrane active peptides [36,37,38], interact with a membrane partner(s) that is mainly expressed in the membrane of transformed cells. Upon binding, the helical structure of RT33 and RT33DR could allow the formation of pores in the cancer cell membrane, as observed with other membranolytic, pore forming peptides [39]. Identification of RT33 and RT33DR membrane partner(s) is currently underway. The potential use of RT33 and RT33DR as novel anticancer drugs was then evaluated in the context of the Sézary syndrome, a leukemic and aggressive form of cutaneous T cell lymphoma (CTCL) with poor prognosis. We chose to focus on Sézary syndrome because current treatment options are limited, emphasizing the need for novel agents and therapeutic targets in these patients [40]. The treatment of primary patient-derived samples with either RT33 or RT33DR, but not the hAP10 or hAP10DR shuttles alone, induced selective death of malignant T cell clone, while sparring the non-transformed T cell and the non-T cell populations. As observed with cancer cell lines, RT33 and RT33DR-induced Sézary cells death was necrotic, as validated by 7-AAD staining. In a xenograft model with HUT78 cells, systemic injection of RT33 and RT33DR resulted in a significant reduction in tumor growth, as confirmed by reduced tumor volume. Histological analysis of tumors derived from RT33 and RT33DR treated mice indicated increased necrotic cytotoxicity compared to controls. Although probably not statistically significant, RT33 appears to perform slightly better than RT33DR in our murine Sézary models. This difference might be due to various in vivo parameters, such as peptide stability as well as biodistribution and pharmacokinetic behaviors. Further studies pertaining to RT33 and RT33DR pharmacokinetic and pharmacodynamic properties and in vivo tissue distribution will be necessary to precisely compare the peptides’ intrinsic characteristics. In summary, we have developed novel, short, human-derived, non-cytotoxic and non-antigenic cell permeable peptides, showing excellent cell penetrating ability. Importantly, fusion peptides consisting of the survival protein AAC-11 residues 377–399 linked to the C-terminus of hAP10 or hAP10DR exhibited remarkable anticancer properties both ex vivo and in a mouse model of Sézary syndrome. Therefore, we expect that the unique characteristics of hAP10 and hAP10DR will allow their use for a wide variety of in vitro and in vivo applications.

## 4. Materials and Methods

### 4.1. Materials

All chemicals were purchased from Sigma. All peptides were synthesized by Proteogenix (Strasbourg, France) and guaranteed more than 95% pure by HPLC and mass spectrometry analysis. Peptides sequences are the following: hAP10: RSRSRDRRRK; hAP10DR: RSRSRDRRRK; RT33: RSRSRDRRRKLQYFARGLQVYIRQLRLALQGKT; RT33DR: RSRSRRRRRKLQYFARGLQVYIRQLRLALQGKT.

### 4.2. Patients and Cells

SS diagnosis was established on recognized international clinical, histological, and biological criteria. Blood from SS patients with more than 90% of malignant CD4^+^ T cells (identified by their TCR-Vβ rearrangement) was collected for the present study, which was approved by the institutional ethics committee (Saint Louis Hospital, Paris. Protocol no. 2011-oct.12735). Peripheral blood mononuclear cells (PBMC) were isolated from heparinized venous blood by density gradient centrifugation over lymphocytes separating medium (LSM; PAA Laboratories, Les Mureaux, France). The Sézary cell line HUT78 was amplified as previously described and cultured in RPMI 1640 supplemented with 2 mM L-glutamine, 1% penicillin-streptomycin (Invitrogen) and 10% heat-inactivated fetal calf serum (Life Technologies). All other cell lines were cultured in DMEM supplemented with 2 mM L-glutamine, 1% penicillin-streptomycin (Invitrogen) and 10% heat-inactivated fetal calf serum (Life Technologies).

### 4.3. Peptides Characterization

The support vector machine (SVM)-based prediction of cell penetrating properties was performed with the online CellPPD tool [41]. Secondary structure predictions were performed with PSIPRED [25]. Three-dimensional structure predictions were carried out with I-TASSER [26]. Figures were generated with PyMOL (Schrödinger, Available online: http://www.schrodinger.com). Energy maps of the peptides were analyzed and generated using Molegro Molecular Viewer.

### 4.4. Cellular Uptake Quantification

Cellular internalization of FITC-labelled peptides was analyzed using flow cytometry. Cells were incubated in the presence of the peptides (5 µM each) in complete medium for 1 h. Cells were then washed three times in PBS and incubated with trypsin (1 mg/mL) for 10 min to remove the extracellular unbound peptides. Finally, cells were suspended in PBS and kept on ice. FITC fluorescence intensity of internalised peptides in live cells was measured by flow cytometry using BD FACS CANTO II^TM^ by acquiring 1 × 10^4^ cells. Data was obtained and analysed using FACSDiva^TM^ (BD biosciences) and FowJo software (Treestar Inc., Ashland, Oregon, USA). In some experiments, cellular internalization was analysed using multimode spectrophotometry. Briefly, after incubation with the FITC-labelled peptides, cells were washed as described, centrifuged and the cell pellet resuspended in 300 µL of 0.1 M NaOH. Following 10 min incubation at room temperature, the cell lysate was centrifuged (14,000 × g for 5 min) and the fluorescence intensity of the supernatant determined (494/518 nm). The fluorescence of the cellular uptake is expressed as fluorescence intensity per mg of total cellular protein.

### 4.5. Live Cell Microscopy

U2OS or C8161 cells (2 × 10^4^) were seeded into Lab-Tek II chamber slides (Nalgen Nunc, Rochester, NY, USA). Then, 48 h later, cells were incubated with either FITC-labelled peptides (5 µM) or the studied EGFP fusion recombinant proteins (5 µM) in complete medium for 1 h at 37 °C. Following incubation, the cells were washed three times in PBS and imaged using a Zeiss Axiovert 200 M inverted fluorescence microscope.

### 4.6. Cell Viability and Lactate Dehydrogenase (LDH) Release Assays

Cells survival was assessed with the CellTiter 96^®^ Aqueous One Solution Cell Proliferation Assay kit (Promega, Madison, WI, USA). Necrotic plasma membrane permeabilization was assessed by lactate dehydrogenase (LDH) leakage in the culture medium with the CytoTox 96^®^ Non-Radioactive Cytotoxicity Assay kit (Promega, Madison, WI, USA).

### 4.7. Hemolysis Assay

Mice blood was centrifuged at 2000 rpm for 10 min. Red blood cell pellets were washed five times with PBS and resuspended in normal saline. For each assay, 1 × 10^7^ red blood cells were incubated with or without peptide (30 µM) in normal saline at 37 °C for 1 h. The samples were then centrifuged and the absorbance of the supernatant was measured at 540 nm. To determine the percentage of lysis, absorbance readings were normalized to lysis with 1% Triton X-100.

### 4.8. Immunogenicity Assay

RAW 264.7 murine macrophages were seeded (1 × 104 cells/cm^2^) in a 24-well plate and allowed to grow for 24 h. Then, cells were left untreated or exposed to the hAP10 or hAP10 DR peptides (10 µM) or to LPS (*E. coli* O111: b4, 1 µg/mL) as a positive control for 24 h. Levels of IL-6 in the supernatants were analyzed using an Mouse IL-6 Quantikine ELISA Kit (R&D system, Minneapolis, MN, USA).

### 4.9. Recombinant Protein Purification

TAT, penetratin, hAP10, and hAP10 DR nucleotide sequences with EGFP inserted at the C-terminal end were subcloned in the pET-21 a vector system (Novagen) and the constructs used to transform *E. coli* BL21(DE3) cells (Invitrogene). The transformed cells were grown at 37 °C in LB broth containing 100 ug/mL of ampicillin to an A_600_ of 0.6 and induced with 1 mM IPTG for 3 h at 30 °C. After harvest, the cells were resuspended in ice-cold Lysis buffer (20 mM HEPES, 100 mM NaCl, 10 uM ZnSO_4_, 1 mM Tris-HCl, pH 8.0) containing proteases inhibitors and lysed using a French press. Cell lysates were centrifuged at 4 °C for 30 min at 45,000 rpm. Ni/NTA affinity purification was performed on an AKTA fast protein liquid chromatography (FPLC) system using 2 mL HisTrap HP columns (GE Healthcare Biosciences Uppsala, Sweden) equilibrated in wash buffer (20 mM HEPES, 100 mM NaCl, 10 uM ZnSO_4_, 1 mM Tris-HCl, 20 mM imidazole, 10% glycerol, pH 8.0). Bound proteins were eluted using elution buffer B (20 mM HEPES, 100 mM NaCl, 10 µM ZnSO_4_, 1 mM Tris-HCl, 300 mM imidazole, pH 8.0). Fractions were collected and analysed by Coomassie staining to assess purity.

### 4.10. Flow Cytometry Analysis of Sézary Patients’ Cells

PBMC exposed or not to RT33 or RT33 DR were processed for flow cytometry to assess cell death. Cells were labelled with a mix of anti-TCR-Vβ-FITC, -CD3-PE, and -CD4-PE-Cy7 mAbs (Beckman Coulter). Detection of apoptotic cells was performed using 7 AAD (BD Biosciences). Cells were analyzed on a CytoFlex cytometer (Beckman Coulter) and data treated with FlowJo software.

### 4.11. Xenograft Tumor Model

Animal experiments were approved by The University Board Ethics Committee for Experimental Animal Studies (#2303.01). Xenograft tumors were obtained by subcutaneous injection of 10^6^ HUT78 cells in the right flank of 8-week-old female NOD-SCID-gamma (NSG) mice, bred and housed under pathogen-free conditions at our animal facility (IUH, Saint Louis Hospital, Paris, France). Treatment started after randomization when tumors were visible and consisted of daily intraperitoneal (i.p.) injection of normal saline or RT33 or RT33 DR in normal saline (*n* = 5 per group). Tumor volume was measured every other day and calculated as: long axis × short axis^2^ × 0.5. Animals were euthanized after 21 days of treatment or when tumor size reached the ethical end point and visceral organs were excised for a gross pathological examination. Tumors were fixed in 4% neutral buffered formalin and embedded in paraffin. Sections (4 µm) were stained with hematoxylin-eosin (H&E) and subjected to microscopic analysis and image recording. Histological analysis was performed at the HistIM facility of Cochin Institute (Paris, France). Slides were imaged using a Lamina multilabel slide scanner (Perkin Elmer).

### 4.12. Statistical Analysis

Statistical analyses were performed using a –Whitney or Student *t* test. *p* values of less than 0.05 were considered statistically significant. Data are expressed as mean ± SD.

## 5. Conclusions

In conclusion, we disclosed new cell penetrating sequences, hAP10 and hAP10 DR, that exhibit excellent cell penetration efficiencies, alone and as cargo protein carriers, in both normal and cancerous cells, equalling or surpassing classical CPPs, such as TAT and penetratin, while being among the shortest CPPs identified thus far.

Based on these results, we designed two anticancer peptides, termed RT33 and RT33 DR, consisting of residues 377–399 of the survival protein AAC-11 attached to the C-terminus of hAP10 or hAP10 DR, respectively, and report the proof-of-principle experiments for testing the therapeutic value for using RT33 and RT33 DR as novel treatment agents for Sézary syndrome. Our data show that the obtained peptides were able to induce tumor cells, but not normal cells, death both ex vivo on Sézary patients’ circulating cells and to inhibit tumor growth in vivo in a sub-cutaneous xenograft mouse model for the Sézary syndrome.

## Supplementary Materials

The following are available online at https://www.mdpi.com/2072-6694/12/7/1858/s1, Figure S1: Fluorescence quantification of FITC-labelled hAP10 and hAP10 DR uptaken in U2 OS and C8161 cells. Cells were incubated with 5 µM of FITC-labelled hAP10 and hAP10 DR or penetratin and TAT as controls for 1 h in complete medium, washed with PBS and the fluorescence of the cell lysis measured as described in Material and Methods. Data are means ± s.e.m. (*n* = 3); Figure S2: Internalization mechanisms of hAP10 and hAP10 DR. U2 OS cells pre-incubated at 4 °C or with heparin sulfate (20 μg/mL), sodium azide (0.1%), CPZ (50 µM), MBCD (1 mM) or EIPA (50 µM) for 30 min or left untreated were incubated with 5 µM of FITC-labelled hAP10 and hAP10 DR for 1 h in complete medium. Cells were then washed with PBS, detached with trypsin, washed and suspended in PBS, then subjected to flow cytometry (left). Right: Bar diagram representing the uptake of the FITC-labelled peptides as mean cellular fluorescence from the flow cytometry analysis of live cells positive for FITC. Data are means ± s.e.m. (*n* = 3); Figure S3: hAP10 and hAP10 DR-mediated delivery of a GFP cargo into cells. U2 OS cells were exposed to the indicated GFP fusion proteins (5 µM) for 1 h. Cells were then fixed, stained for DNA, and examined by fluorescence microscopy. The scale bar indicates 10 µm; Figure S4: HUT78 cells were exposed to 20 µM of RT33, RT3 DR or a peptide comprising AAC-11 residues 377 to 399 for 20 h. Viability was then assessed by an MTT assay. Data are means ± s.e.m. (*n* = 3); Figure S5: U2 OS cells were exposed to increasing concentrations of RT33 or RT3 DR for 20 h in the presence and absence of 50 μM zVAD-fmk or 50 μM Necrostatin-1 (Nec-1). Viability was then assessed by an MTT assay. Data are means ± s.e.m. (*n* = 3).
